# Sorption Processes of Selected PAHs on Selected Fire-Resistant Materials Used in Special Firefighter Clothing

**DOI:** 10.3390/ma17081741

**Published:** 2024-04-10

**Authors:** Anna Rabajczyk, Justyna Gniazdowska, Piotr Stojek, Łukasz Bąk

**Affiliations:** Scientific and Research Centre for Fire Protection-National Research Institute, Aleja Nadwiślańska 213, 05-420 Józefów, Poland; jgniazdowska@cnbop.pl (J.G.); pstojek@cnbop.pl (P.S.); lbak@cnbop.pl (Ł.B.)

**Keywords:** special firefighter’s clothing, aramid fibers, PAHs, fires

## Abstract

Fires constitute a significant threat due to the pollutants emitted and the destruction they cause. People who take part in firefighting operations must be equipped with appropriate tools, including special clothing that will allow them to work and guarantee safety. One of the threats is represented by compounds from the PAH (*Polycyclic Aromatic Hydrocarbons*) group, which are characterized by high toxicity and carcinogenicity. Therefore, it is important that the materials used constitute a barrier to contamination. Various materials from which individual elements of special firefighter’s clothing are made were tested. Additionally, the effect of height on the possibility of sorption of PAH compounds on a given type of material was analyzed. Based on the obtained analysis results, it was found that both the type of material and the zone in which the clothing items are used are important in the sorption processes of pollutants. For example, PAHs with high molecular weight are most likely to settle on rubber, i.e., the material from which shoes are made, with the exception of Chrysene, whose presence was found primarily in aramid fibers, i.e., the material from which trousers and jackets are made. However, among PAHs with low molecular weight, compounds such as Methylnaphthalene,1- and Fluorene were sorbed on the rubber surface in large quantities. The only compound that is present in comparable amounts in all materials is Acenaphthylene. Data in this area may be important for taking further actions related to the modification of materials used in special fire brigade clothing and in their cleaning processes.

## 1. Introduction

**Figure 1:**
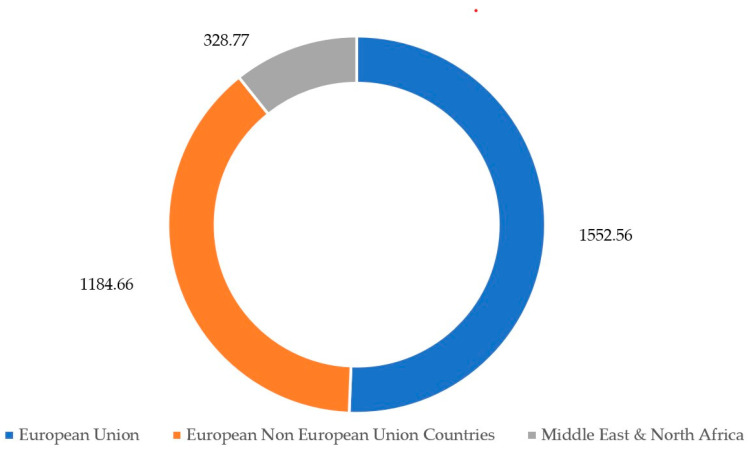
Average annual number of fires in 2020–2023 covering 30 ha or more (based on [2]).

Fires are an integral part of life, which makes the probability of their occurrence very high [1]. The average annual number of fires over 30 ha or more in the European Union (EU) in 2020–2023 was 1552.56, in European countries outside the European Union 1184.66, and in the Middle East and North Africa 328.77 [2] (Figure 1). In Poland, during the same period, the average annual number of all fires, regardless of size, was 117,618 [3]. It should also be added that in the United States (USA), in the years 2020–2023, the number of fires, only in forest areas, was on average 59,704 [4], while over the last 20 years, in the western part of the USA, there has been an increase in both the frequency and size of forest fires [5].

**Figure 2:**
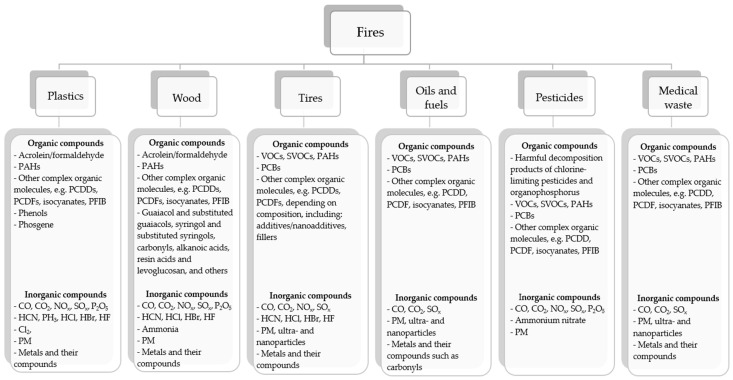
Examples of substances emitted during a fire from various materials (where: PAHs—*Polycyclic aromatic hydrocarbons, VOCs*—volatile organic compounds, *SVOCs*—semi-volatile organic compounds, PCDDs—polychlorinated dibenzodioxins, PCDFs—polychlorinated dibenzofurans, PFIB—perfluoroisobutene, PM—particulate matter, PCBs—polychlorinated biphenyls).

The increase in the number of fires in the world is mainly caused by anthropological interference in the environment. It should be noted that each fire is different (Figure 2), and therefore the composition of compounds emitted to the environment varies [6,7,8,9,10,11,12,13,14,15,16].

The size and composition of substance emissions into the environment are determined primarily by the type and moisture of the fuel burned (materials, building structure elements, type of forest, etc.) and the fire temperature [6,7,8,9,10,11,12,13,14,15,16]. Emitted compounds may significantly affect human health, causing, among other things, diseases of the respiratory system and circulatory system, as well as cancer (including kidney, lung and liver cancer caused by ethylbenzene) [17,18,19,20,21,22,23,24].

Although the amount of compounds emitted from one event, e.g., a forest fire, may not reach a toxic level, a long-term perspective should be taken into account due to the increasing fire seasons, larger areas, or the diversity of elements covered by the fire [2]. The emitted substances often belong to the group of substances that are persistent in the environment, and may bioaccumulate. It should also be added that when extinguishing fires, the threat is not only the substances emitted, but also, depending on the type of fire, radiant or convective heat, explosions, falling objects, debris, small particles floating in the air, limited inflow oxygen, hot liquid, molten substances, and noise [25,26,27]. Taking into account the variety of threats to which firefighters are exposed, it is necessary to have appropriate tools to increase their safety during the implementation of activities [2]. One of them is protective clothing, i.e., a special clothing product that provides protection for the torso, neck, arms and legs of firefighters, excluding the head, feet and hands [26,27,28]. In the case of chemical and/or gas cleaning activities, it is necessary to use clothing appropriate for this type of activity [29]. To completely protect firefighters from hazards, it is necessary to use additional personal protective equipment protecting the face, hands and feet, and, if necessary, respiratory protective equipment [21]. It is also crucial to find a balance between thermal protection and comfort (e.g., avoiding heat stress) and other potential requirements [20]. The clothes, which consist of two basic elements, i.e., a jacket and trousers, are produced in multi-layer form. Each layer is characterized by appropriate protective properties and different functionality, with the first layer being the most durable element of the special clothing. This layer protects against flames, thermal radiation, external chemicals, water, abrasions, cuts and wounds, but the strength and properties of the material depend on its composition and thickness. In clothes available on the market, the first layer is usually synthetic aramid (Nomex, Kevlar), polyamide (Kermel), polyimide (Lenzing) or polybenzimidazole (PBI) fibers. Impregnated cotton fabrics can also be found, but they are the least frequently used [26,27,28].

The second layer acts as an insulator against the penetration of water, chemicals and pathogens from the outside. It also plays a key role in the breathability and insulation of the entire garment, which is why it is often called a moisture barrier. The material used for this layer is a hydrophobic microporous membrane, usually made of plastics such as polyurethane, polyester or polytetrafluoroethylene. One of the most frequently used materials in firefighter clothing is Goretex [26,27,28,29,30]. The third, thermally insulating layer, thanks to the use of airbags and climate micro-chambers, is designed to ensure wearing comfort and serves as a thermal barrier. It minimizes thermal stress and removes moisture from the body surface. It is made of aramid, aramid–viscose and polyester fibers. The effectiveness of the layer is determined by the heat transfer rates from the flame and thermal radiation [23]. Firefighting gloves, which, in addition to high protection, must provide the user with comfortable wearing while maintaining full efficiency and grip, have a layered structure, just like the jacket and trousers. The inner layer consists of a lining ensuring comfort of use and a thermal barrier. The middle layer is a membrane that protects against chemical threats and ensures the one-sided permeability of gases and moisture. The third one, which is the outer layer, is a barrier resistant to cuts, abrasion and flame, while ensuring adhesion to smooth and wet surfaces [31,32].

Firefighters’ footwear is also an important element of protection, and has many important functions depending on the situation. Its correct selection is crucial to ensuring the user’s safety, comfort and protection. According to the EN 15090:2012 standard, firefighting footwear is divided into two classes: class I—leather or fabric footwear with only plastic elements and class II—all-rubber or all-plastic footwear [33]. The above classes can be divided into three types depending on the conditions in which they will be used:

- Type 1—for outdoor operation, including operation in a forest fire environment, no puncture protection and finger protection, no protection against chemical hazards;
- Type 2—for rescue and firefighting activities, puncture protection and finger protection, no protection against chemical hazards;
- Type 3—for all types of activities, puncture protection, finger protection and protection against chemical agents.

For this reason, high-quality, heat- and waterproof natural leather (calf, cow, lamb, kid, sheep, pig), pig suede, goat suede or rubber materials (such as vinyl) are used to produce specialized footwear. Their ability to breathe and air permeability are also important. The interior of specialized shoes is usually made of a removable cotton–polyester lining [31,32,34].

It should be noted that firefighter protective clothing must meet the design and use requirements specified in the standards, including: ISO 11613 [35] or ISO 11999-3 [36], EN 469 [37] and NFPA 1971 [38], Ref. [1]. The requirements apply to clothing as a whole, i.e., jacket and trousers, and other items of equipment such as shoes, helmets or gloves. Research conducted so far in various centers focuses on identifying contaminants in firefighters’ clothing, threats to firefighters’ lives and health, and the possibility of cleaning clothes in the washing process. The aim of this work was to determine the amount of selected organic compounds of a toxic nature from the PAH group that is adsorbed on firefighter’s clothing in relation to (I) the type of material from which individual elements of the special clothing are made and (II) the height of sampling from the clothing, and determining the zones most exposed to the adsorption of compounds. The information obtained can then be translated into the proper handling of clothing and the minimization of the impact of these compounds on the firefighter, as well as increased safety.

## 2. Materials and Methods

### 2.1. Materials and Equipment

Reagents of analytical purity or higher were used in the work. In total, 18 standard carcinogenic PAHs (naphthalene 1000 μg/mL, acenaphthalene 2000 μg/mL, 1-methylnaphthalene 1000 μg/mL, 2-methylnaphthalene 1000 μg/mL, acenaphthene 1000 μg/mL, fluorene 200 μg/mL, phenanthrene 100 μg/mL, anthracene 100 μg/mL, fluoroanthene 100 μg/mL, pyrene 100 μg/mL, benzo(a)anthracene 100 μg/mL, chrysene 100 μg/mL, benzo(b)fluoroanthene 100 μg/mL, benzo(k)fluoroanthene 100 μg/mL, benzo(a)pyrene 100 μg/mL, dibenzo(a,h)anthracene 200 μg/mL, benzo(g,h,i)perylene 200 μg/mL, and indeno(1,2,3-cd))pyrene 100 μg/mL) in a 1:1 mixture of dichloromethane and methanol, CRM quality and PAH assay Mix 3 were supplied by Merck Life Sciences. Solvents (acetonitrile, hexane, dichloromethane, methanol) were provided by Avantor Performance Materials Poland S.A., Gliwice, Poland. Desorption was performed using Supelco tubing of ¼ inch outer diameter and 3.5 inch length, supplied by Merck Life Science Sp. z o.o., Poznań, Poland.

Special firefighter’s clothing, including a jacket and trousers, according to the manufacturer’s declaration, consisted of (% by weight):

- Outer layer—99% aramid fiber and 1% antistatic fiber;
- Middle layer—aramid fibers (65%) covered with a polyethylene film (35%);
- Inner layer—two-layer lining, first viscose layer (50%) second aramid layer (50%).

This type of clothing has been chosen for this study due to the fact that the majority of special firefighting clothing that has received a Polish certificate of admittance after being tested in our Institute utilizes this type of fibers.

The shoes used for analysis were of class 0 type 2 and were made of rubber with a cotton lining. Firefighting gloves (type RGS-355) made of full-grain cowhide with a flame-retardant finish were also used for the tests. These were protected against water seepage with a HIPORA polyurethane membrane. Additionally, the gloves had an inner insert made of Kevlar fibers, providing additional protection against mechanical and thermal threats. They had 3M reflective tape on the back part. All equipment was repeatedly exposed to fire conditions during extinguishing (i.e., in 25 fires of various types, including wood and liquid fuel fires) and was not subject to cleaning. In order to conduct qualitative tests of the samples, a gas chromatograph coupled with mass spectrometer Shimadzu GC-MS QP 2010 Ultra SE and a TD-30 thermal desorption apparatus purchased from Shimadzu Corporation, Kyoto, Japan were used. BIP+ helium with a purity of >99.99997% was used as a carrier gas.

### 2.2. Sampling Methodology

**Figure 3:**
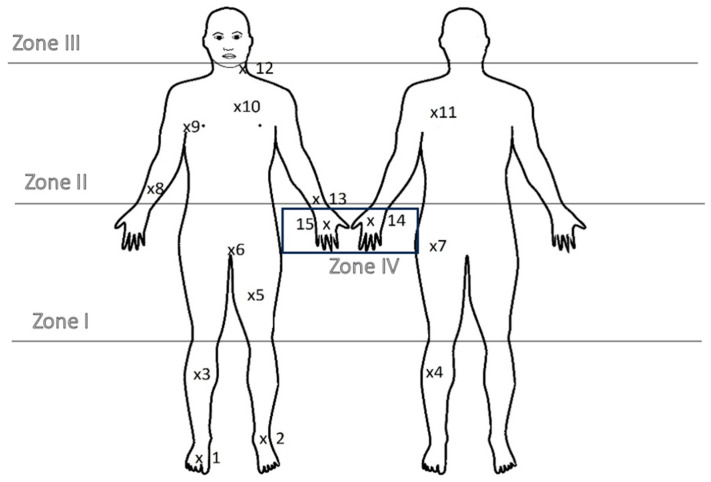
Scheme of collecting samples from special firefighter’s clothing (own work). 1—toe of shoe; 2—shoe insole; 3—knee from the front; 4—knee from the back; 5—thigh from the front; 6—crotch; 7—buttock; 8—inner cuff; 9—armpit; 10—chest in front; 11—back at shoulder blade height; 12—inner collar cuff; 13—inner cuff from the forearm; 14—gloves—outside; 15—gloves—inner side.

Clothing, including shoes, trousers, jacket and gloves, was divided into four zones (Figure 3):

- Zone I—shoes, i.e., from the ground to the knees;
- Zone II—trousers, i.e., from the knees to the waist;
- Zone III—sweatshirt, i.e., from the waist to the neck;
- Zone IV—gloves.

Here, 15 sampling locations were designated (Figure 3), which take into account differences in height and materials used in individual parts of the clothing.

**Figure 4:**
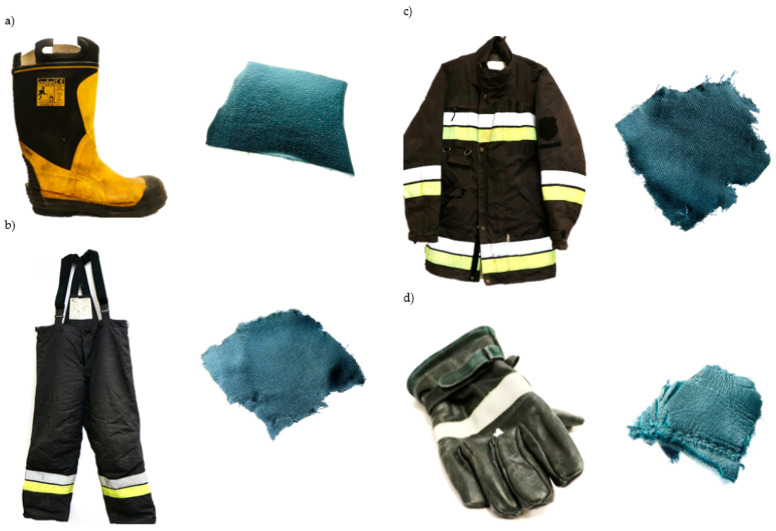
Material samples from shoes (**a**), trousers (**b**), jacket (**c**) and gloves (**d**).

Clothing samples weighing (Figure 4) approximately 1–3 g were taken from special firefighter clothing using Agar Scientific Ltd. (Stansted, Great Britain, UK) biopsy samplers, and then they were placed in desorption tubes. Three samples were taken from each place and the obtained analysis results were averaged.

### 2.3. Preparation of the Calibration Curve

The QTM PAH 3 analytical standard was diluted in the ratios of 1:100, 1:500, 1:1000, 1:5000, 1:10,000 and 1:100,000, as described in “Organic Preparation” A.I. Vogel [39]. Then, the appropriate amount of solvent was taken from the solutions prepared in this way to prepare standard curves. The contents of analytes in the sorption tubes prepared in this way were 10, 20, 100, 200 and 1000 ng, respectively. The ca libration curve was plotted by plotting the relationship between the obtained surface areas on the ordinate axis and the theoretical content of a given polycyclic aromatic hydrocarbon in the tube. Each standard concentration was prepared three times. Then, the dependence of the response (number of secondary ion counts) on concentration was determined for each analyte, and the standard curves and relative standard deviation (RSD) of the calibration curve points were calculated automatically using the gas chromatograph software GC-MS solution Version 4.45 SP1 built 25.09.2017. The parameters of the obtained curves are presented in Table 1.

### 2.4. Chromatographic Analysis with Thermodesorption

**Figure 5:**
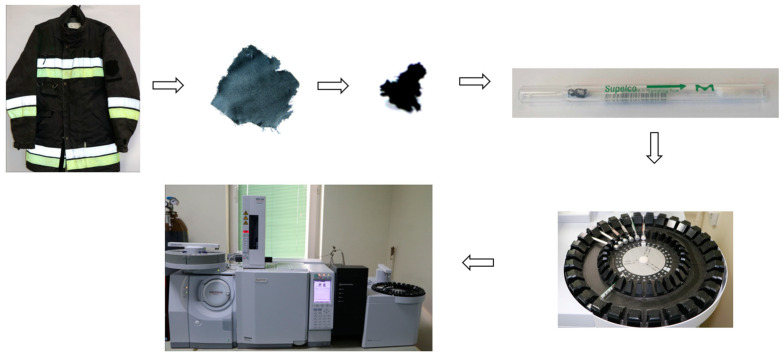
Diagram of the analytical procedure.

The collected material samples were placed in desorption tubes and heated in a thermodesorber to a temperature of 350 °C. Various desorption and trap adsorption temperatures have been tested, and it was determined that the best signal to noise ratio was obtained on the highest possible desorption temperatures recommended by thermodesorber manufacturer. The process was carried out under the best established conditions, in accordance with the parameters presented in Table 2 and according to the diagram shown in Figure 5.

Chromatographic analyses were performed using a gas chromatograph with a secondary ion mass spectrometer and a GC-MS-TD thermal desorption apparatus from Shimadzu. The samples were subjected to chromatographic analysis using a Zebron ZB PAH capillary column obtained from Phenomenex, Torrance, CA, USA with a length of 30 m, a diameter of 0.25 mm and a film thickness of 0.25 µm. The carrier gas was helium (flow 0.92 mL/min). A temperature program was used for the analyses, the parameters of which are presented in Table 3.

The uncertainty of the determined results is expressed as the relative standard deviation (RSD), calculated for the points of the calibration curve. Analyses for each analyte were repeated three times. The limit of quantification (LOQ) value for each analyte was expressed as 10 times the standard deviation value of the blank chromatogram baseline disturbance. The calculated LOQ value for each analyte was less than 0.02 μg/g. Therefore, this LOQ value was assumed for all analytes.

## 3. Results

Analyses of samples of materials taken from firefighters’ special clothing were carried out at the Laboratory of Fire Extinguishing Equipment and Agents, accreditation number AB060. As a result of the work carried out, the presence of the analyzed PAH compounds in the analyzed clothing items was found in varying amounts (Table 4).

**Figure 6:**
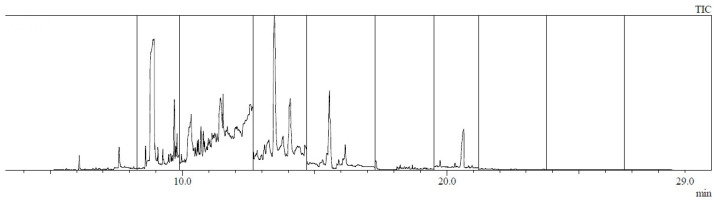
Chromatogram of a sample taken from the outer layer of the shoe (sample no. 1: toe of the shoe).

**Figure 7:**
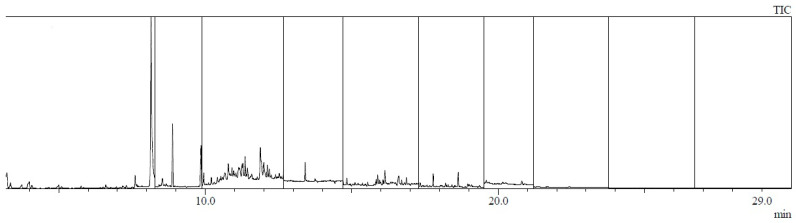
Chromatogram of a sample taken from the trousers at the level of the buttock (sample no. 7: buttock).

**Figure 8:**
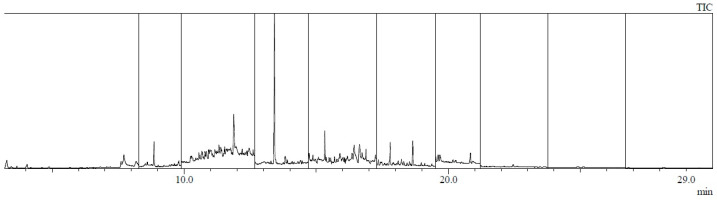
Chromatogram of a sample taken from the jacket in the armpit crease (sample no. 9: armpit).

**Figure 9:**
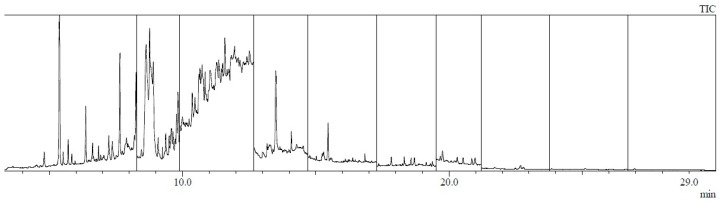
Chromatogram of a sample taken from the outer layer of the glove—palm (sample no. 15: gloves—inner part).

Example chromatograms of material samples analyzed for the content of PAH compounds are presented in Figure 6, Figure 7, Figure 8 and Figure 9.

## 4. Discussion

Compounds emitted during a fire exhibit various physicochemical properties, depending on their capacity for accumulation. Whereas low-molecular-weight compounds and inorganic gases easily undergo diffusion and do not have a tendency to accumulate on fibers, high-molecular-weight compounds with corresponding higher boiling points have the ability to accumulate on clothing fibers, and their evaporation process might be slow. Moreover, low-molecular-weight organic pollutants usually do not form in amounts similar to their corresponding PEL values (Permissible Exposure Limits), which makes monitoring them in special clothing more difficult, and means that adverse effects after long-term skin exposure are not as significant as the effects of exposure to PAH compounds. The level of other harmful chemicals that could be present in firefighters’ special clothing after production processes, like plasticizers and fire retardants, i.e., phthalates and the organophosphorus esters and sealants used to maintain good barrier properties (usually based on perfluoroalkyl substances (PFAS)), may also have some level of impact on human health, but the concentration of these contaminants is expected to be the highest directly after manufacturing. Therefore, we chose to examine in this study only the PAH content in the outer layer of firefighting special clothing.

Chemical substances deposited on clothing can penetrate the structure of the material and then permeate through subsequent layers of materials [40]; as a result, this may lead to the direct contact of chemical substances with the skin, especially the hands, neck and back [41,42]. PAHs are a large group of chemical substances containing from two to seven conjugated aromatic rings in their structure. The simplest representative of this group of compounds is naphthalene, consisting of two benzene rings with a boiling point under standard conditions of 218 °C [43]. These compounds occur in the environment as a result of the incomplete combustion of coal from natural and anthropogenic sources, including: industry, motor vehicle engines, and furnaces in residential buildings. These compounds are characterized by various properties that determine their ability to accumulate in the environment, their reactivity, and their participation in biochemical or physical processes. As a result of the conditions prevailing during fires, they may be deposited on surfaces in the cooling zone of fire gases [44]. Since these compounds always occur in the form of a mixture of components, the concentration of which is strongly correlated with the conditions of the combustion process, fluctuations in these concentrations can also be used to identify the source of their production [45]. Literature data [46] show that PAHs containing up to three aromatic rings occur primarily in the form of gases, while for PAHs from pyrene, the main forms of their occurrence are solid particles, the share of which increases up to 100% in the case of perylene and indeno(1,2,3-cd)pyrene [47].

All these compounds are easily sublimating solids with high vapor pressure, which affects their ability to migrate and absorb on various materials. Low-molecular-weight PAHs occur in the atmosphere primarily as vapors, while higher-molecular-weight PAHs form aerosols by condensation after emission. This property, combined with the possibility of migration as a result of the creation of a chemical equilibrium between the adsorbed PAHs and their vapors, affects their ability to migrate deep into the material, which, combined with their hydrophobic nature, allows them to penetrate the skin and mucous membranes. For this reason, these compounds are identified as one of the main sources of firefighters’ exposure to toxic chemical compounds outside the immediate vicinity of areas and facilities affected by the fire [47,48,49]. For this reason, many studies have been carried out to determine the adsorption capacity of various pollutants, including PAHs, on fabrics used in clothes, also taking into account the type of finishing. It has been shown that, among others, hydrophobic finishes increase the amount of PAHs adsorbed on outdoor fire brigade fabrics based on meta- and para-aramid, and fabric with a single layer was characterized by a high PAH adsorption capacity [50]. A significant difference in the PAH adsorption rate was also observed, especially between single- and double-layer fabrics. The amounts of 10 analyzed compounds from the PAH group (i.e., acenaphthene, acenaphthylene, anthracene, benzo[a]anthracene, benzo[a]pyrene, benzo[b]fluoranthene, benzo[e]pyrene, benzo[g,h,i]perylene, benzo[j]fluoranthene, benzo[k]fluoranthene, chrysene, dibenzo[a,h]anthracene, fluoranthene, fluorene, indeno[1,2,3-c,d]pyrene, naphthalene, phenanthrene, pyrene) were higher in single-layer fabric than in two-layer fabrics, while the total PAH contamination was 77% higher in the single-layer fabric than in the two-layer fabric [51]. In the case of a material consisting of three layers of fabrics based on meta- and para-aramids, it was shown that soot particles and some PAHs were trapped in the outer layer, but most of the PAHs moved towards the moisture barrier layer along the pores of the outer fabric under the influence of high temperature. Polycyclic aromatic hydrocarbons were the most abundant in the middle layer, reaching 228.9 mg/kg, while in the outer and comfort layers (i.e., inner, closest to the skin), they were 68.8 and 0.9 mg/kg, respectively. The work also showed that the glove moisture barrier could prevent PAHs from penetrating between the moisture barrier layer and the inner layer.

Aramids belong to the group of fiber-forming polyamides. Their characteristic feature is the presence of aromatic groups in their main chains. Some aramids contain only aromatic groups between the amide bonds, while others also contain aliphatic groups. The more aromatic groups there are in the structure, the greater the mechanical, thermal and fire resistance of the material. However, the solubility decreases, which causes difficulties in processing the material. The system of successive aromatic groups and amide bonds creates a structure of high stiffness due to the delocalization of electrons from the π orbitals of aromatic systems to amide bonds, i.e., −C(=O)−NR_2_ (R–H, various functional groups). Rigid polymer chains crystallize more easily and arrange themselves more easily during spinning, thanks to which the obtained fibers have a very ordered microstructure. The most famous aramid from the para-aramid group (poly(terephthaloyl terephthalamide, PPTA) is poly(phenylene-1,4-diamide) (known as Kevlar), and from the meta-aramid group (polymetaphenylene isophthalamide PMTA) it is poly(phenylene-1,3-diamide) (known as Nomex) [52,53,54,55]. Single Kevlar fibers tested in laboratory conditions have a tensile strength of 3620 MPa. This material retains its properties in the temperature range from −200 °C to 245 °C. When in contact with fire, Kevlar does not melt or drip, but at a temperature of approximately 500 °C it decomposes without releasing toxic substances. The properties of Nomex are similar to those of Kevlar, but it has a lower softening temperature (approx. 220 °C) and decomposition temperature (approx. 350 °C), than Kevlar and dissolves more easily in H_2_SO_4_. Generally speaking, aramids are also substances that are very resistant to most chemicals, such as acids, bases and solvents. However, they degrade due to the action of ultraviolet rays, which is why they are rarely used outdoors without protection against sunlight [52,53,54,55]. The aramid bond is characterized by hydrophilicity, which leads to the absorption of moisture by all aramids. However, depending on the preparation method and the obtained structure, they may show differences in this area. For example, at a temperature of 20 °C and a relative humidity of 55%, the Kevlar 149 module (density 1.47 g/cm^3^, tenacity 18) absorbs moisture at a level of ~1%, while Kevlar 29 (density 1.43 g/cm^3^, tenacity 20–23) does so at the level of ~7% [55]. Aramid fibers also easily absorb hydrophobic substances, such as paraffin [52,53,54,55,56]. For this reason, aramid fibers are used in the processes of the sorption and extraction of PAHs from solutions [56,57].

There is little information in the literature about the sorption predispositions of individual pollutants on the materials from which firefighters’ clothing is made. Therefore, in this study, 15 samples were collected from selected places (Figure 3) and analyzed for the quantitative assessment of selected compounds from the PAH group, taking into account the types of different materials from which individual parts of the clothes were made.

The materials from which the firefighter’s special clothing is made were analyzed (Figure 4), as follows:

- Jacket and trousers—99% aramid fiber and 1% antistatic fiber (outer layer), 65% aramid fiber covered with a polyethylene film (35%) (middle layer), two-layer lining, including the first viscose layer (50%) and the second aramid layer (50%) (internal layer);
- Shoes—rubber, cotton lining, steel toe;
- Firefighters’ gloves—full-grain cowhide, protected with HIPORA polyurethane membrane, inner insert made of Kevlar fibers.

The analysis of the obtained results confirms the findings in the literature that the highest content of PAHs is found in samples taken from the inner part of a firefighter’s glove and the upper part of a firefighter’s footwear. This may indicate that the dominant factor in clothing contamination is the deposition of dust on surfaces touched by firefighters, while the direct deposition of aerosol on the surface of clothes is much less important. This phenomenon results from the fact that if rescue operations are carried out in an open space during a fire, aerosols and fire gases rise due to the temperature difference. Then, they are partially dispersed in the air and begin a very slow process of descent in areas away from the rescue operation. The main source of pollution is direct contact with the burned surface, ashes, etc.—materials remaining after the fire has been extinguished. It should be noted that the shoes analyzed were made of flame-retardant rubber mixtures applied to a weave of cotton-polyester fabrics. Dust, including soot, may be sorbed on such a surface, which may increase the amount of dirt on the firefighter’s boot. Firefighter’s gloves, however, made of full-grain cowhide protected with a polyurethane membrane, may accumulate PAHs due to the structure of the protective substance. Membranes of this type are used in the processes of removing contamination or the separation of selected pollutants, as well as in extraction processes, e.g., of phenols [58], PAH capture [59] or benzene [60]. The materials used in these parts of the clothes promote the sorption of PAH compounds, and in the case of gloves, they may even migrate into the structure of the protective membrane, which may translate into difficulties in removing contaminants in the process of cleaning the material. In this situation, gloves must be changed frequently, which in turn can generate hazardous waste that requires special disposal methods. The results of research conducted by Krzemińska and Szewczyńska [42] indicate that the most contaminated areas were the legs and sleeves of protective clothing. It should be noted that this work did not specify the type of material from which the clothes were made.

**Figure 10:**
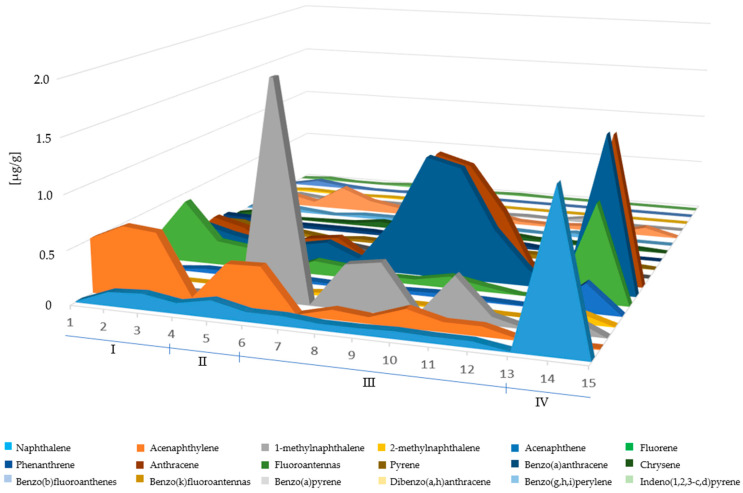
Changes in the amounts of individual analytes in the tested materials with altitude, where 1–15 indicates the sampling point, and I–IV indicates sampling zones.

In the glove samples (Table 4, Figure 10), there was no benzo[a]anthracene, benzo[b]fluoranthene, benzo[a]pyrene, indeno[1,2,3,c,d]pyrene, benzo[g,h,i]perylene or dibenzo[a,h]anthracene, while on the inner side, only 1-methylnaphthalene was observed. In the case of shoes, the most contaminated areas were the toe of the shoe and the front knee, and in both of these places the presence of dibenzo[a,h]anthracene was not recorded. The highest amounts of PAHs were found in samples taken from the toe of the shoe in the case of zone I, from the buttock in the case of zone II, from the armpit in the case of zone III, and from the outside of the glove in the case of zone IV. The amounts of PAHs determined for the materials of jackets and trousers were lower than in the case of rubber from which shoes are made, or grain cowhide protected with a polyurethane membrane in the case of gloves. Indeno[1,2,3,c,d]pyrene, benzo[g,h,i]perylene and dibenzo[a,h]anthracene were not found in the samples from zones III and IV, while the samples from zones I and II had trace amounts of these compounds in their structure (Table 4, Figure 10).

Based on the obtained analysis results, it can be concluded that PAHs with high molecular weight (Table 5, Figure 10) are most likely to settle on rubber, i.e., the material from which shoes are made. The exception is chrysene, whose presence was found primarily in aramid fibers, i.e., the material from which pants and jackets were made.

Among the PAHs with low molecular weight, compounds such as methylnaphthalene, 1- and fluorene were sorbed on the rubber surface in large quantities. The only compound that was present in comparable amounts in all materials is acenaphthylene. Differences in the PAH content in the analyzed samples are therefore a consequence not only of the type of material used, but also of the sampling height.

It should be noted that there are no standards regarding the content of selected PAH compounds for special firefighter clothing. Therefore, the analysis used the OEKO-TEX^®^ guidelines [62], which constitute a global, independent, uniform system for the testing and certification of materials, semi-finished products and textile products at all levels of their production in relation to materials that come into contact with the skin. These guidelines recommend that for class III products, i.e., textiles and materials that do not come into direct contact with the skin, e.g., coats, jackets, outerwear; they also suggest that the contents of the sum of 24 carcinogenic PAHs in the material should be below 10 μg/g. In the case of eight compounds, i.e., benzo[a]pyrene, benzo[e]pyrene, benzo[a]anthracene, chrysene, benzo[b]fluoranthene, benzo[j]fluoranthene, benzo[k]fluoranthene and dibenzo[a, h]anthracene, the value must not exceed 1.0 μg/g [63].

At this moment, there is no holistic publication on firefighters’ special clothing decontamination processes, and the users have to rely only on manufacturers’ instructions. In addition, the outer layer of special clothing is protected by various types of liquid sealants based on polymeric fluorocarbons, which are partially removed during the washing process. Therefore, the direct adoption of clothing decontamination processes from other types of industry that usually require routine washing after every shift, like road paving work, is problematic. This is because recent studies [64] have shown that extensive washing may enable the effective removal of up to 80% of PAH contaminants from the outer layer, but in other studies this effectiveness was lower, or the process was unsuccessful [65]. Cleaning the inner layer is much less effective, probably due to the readsorption of PAHs in this deeper and thicker layers of firefighters’ clothing causing the deterioration of mechanical properties, as first reported by Horn et al. [66]. The effect of the lower cleaning effectivity of the inner layer is probably due to the soaking effect, which prevents the effective removal of washing solution from these layers after the cleaning process. All of these facts contribute to the importance of accurately determining the rate of PAHs accumulation in order to determine optimal wash intervals. This is because after several washings, it is necessary to recreate the sealing layer. However, this process is quite difficult and time-consuming, and involves the risk of exposure to toxic chemicals from the perfluoroalkyl group (PFAS group), some of which are known human carcinogens.

Previous work carried out by various researchers, e.g., Stec et al. [41,48] and Krzeminska and Szewczyńska [42,47,64], has shown the possibility of the accumulation of pollutants in firefighters’ clothes. The threats to life and health of firefighters involved in fire extinguishing and contamination neutralization activities are here analyzed. However, the limit value for the sum of the analyzed PAHs was not exceeded for any of the samples taken from the tested materials. The total content of all PAHs in the material can be estimated by calculating the arithmetic mean content of PAHs in the samples and multiplying it by the weight of the complete firefighting suit, which in the case of the tested suit was 7016 g. This estimate gives a result of approximately 14.79 mg in the clothing, assuming uniform distribution throughout the entire mass of the sample, of which 3.64 mg comprises nine carcinogenic PAHs, in accordance with the definition of the Regulation of the Minister of Family, Labor and Social Policy of 12 June 2018 regarding the maximum permissible concentrations and intensities of factors harmful to health in the work environment [67] (Journal of Laws of 12 June 2018, item 1286, as amended). However, the highest permissible concentration in the air of the working environment for these compounds (which should not worsen the health of an employee exposed to the inhalation of PAHs) is 0.002 mg/m^3^ (expressed as the sum of the products of the concentration of a given compound and its relative carcinogenicity factor). An employee staying in such conditions ventilates the lungs at a rate of approximately 0.093 m^3^/min [68], and the total dose of PAHs absorbed in such conditions will correspond to the absorption of approximately 0.1 mg of benzo(a)pyrene (the relative carcinogenicity coefficient of which is 1). The total sum of the estimated products of the content of carcinogenic PAHs and their carcinogenicity coefficients is approximately 0.32 mg, so it is comparable to the limit values for inhalation exposure.

If a comparison is made with the PEL values (Permissible Exposure Limits) according to OSHA (Occupational Safety and Health Administration) (Table 6), it shows that the estimated content of PAHs in firefighter’s clothing is approximately 25% of the daily permissible exposure in the case of naphthalene, up to 70 times the limit value for anthracene. In the case of the remaining tested compounds from the PAH group, no PEL value was determined [69]. Prolonged exposure to PAH according to many studies [70] is connected to an increased risk of skin, lung, pancreas, bladder, colon, and other types of cancer, as well as many types of cardiovascular diseases, including hypertension and thrombosis. Moreover, because even a low level of exposure might be linked to adverse health effects, including cancers [43]; as such, their further monitoring would be crucial to protect firefighters from occupational hazards.

However, it should be remembered that these estimates assume a uniform distribution of PAHs throughout the sample, which is unlikely considering the high lipophilicity and good barrier properties of materials made of aramid fibers in the case of jackets and trousers. As a consequence, it is also unlikely that these PAHs could migrate to the inner layers of clothing, and consequently also the employee’s skin, at a rate fast enough for them to enter the body at a rate similar to the absorption rate when exposed to limit values of the PAH concentration in air. However, confirmation of this hypothesis requires further research on the distribution of PAHs in individual layers of protective clothing, taking into account the type of material they are made of.

## 5. Conclusions

This study covers protective clothing used by firefighters when fighting fire and undertaking related rescue and firefighting activities. The obtained results indicate that:

- The highest amount of the sum of PAHs in individual clothing items was found in samples taken from the toe of the shoe, and amounted to 5.43 µg/g, while the lowest, amounting to 0.01 µg/g, was found on the inner cuff from the forearm and on gloves—inner side;
- Shoes and gloves are the most contaminated, which is influenced by the type of materials from which these items of clothing are made and the zone of their use;
- In the case of the aramid fiber from which the jacket and trousers were made, the distributions of individual compound were not equal throughout the material, and this is a consequence of the height of sampling;
- The highest amounts of PAHs in the jacket and trousers were found in samples taken from the buttock and armpit, while the lowest were found in samples from the front thigh and inner cuff from the forearm, respectively.

It should therefore be stated that both the type of material and the zone in which the clothing items are used are related to the total amount of PAHs and the presence and amount of individual compounds from the PAH group.

The obtained results indicate that further research is necessary on I) the influence of the type of material on the sorption processes of pollutants emitted during fires, and II) the zone of use of a given item of clothing and the possibility of the sorption of individual pollutants occurring in the fire zone. This information will allow the development of effective solutions to better protect the most endangered areas (e.g., the tip of the shoe, the outer part of the glove, the armpit) from larger amounts of PAH compounds, including the development of, for example, additional protective coatings that limit the sorption of PAH compounds. Moreover, it will be possible to develop solutions for cleaning clothes, and additional methods for the zones most at risk of PAH sorption.

## Figures and Tables

**Table 1:** Parameters of standard curves for individual analytes.

Analyte	Relative Standard Deviation RSD %	Pearson Correlation Coefficient R
Naphthalene	3.05	0.9999
Acenaphthylene	7.75	0.9976
1-methylnaphthalene	7.86	0.9999
2-methylnaphthalene	2.15	0.9999
Acenaphthene	2.76	0.9998
Fluorene	3.66	0.9997
Phenanthrene	11.20	0.9996
Anthracene	4.66	0.9998
Fluoroantennas	5.85	0.9998
Pyrene	6.83	0.9997
Benzo(a)anthracene	9.67	0.9998
Chrysene	12.09	0.9996
Benzo(b)fluoroanthenes	11.76	0.9997
Benzo(k)fluoroantennas	7.61	0.9997
Benzo(a)pyrene	8.26	0.9996
Dibenzo(a,h)anthracene	10.82	0.9985
Benzo(g,h,i)perylene	23.50	0.9983
Indeno(1,2,3-c,d)pyrene	13.06	0.9965

**Table 2:** Parameters for the thermodesorption of analytes from material samples.

Thermodesorber Operating Parameter	Value
Sample weight	1–3 mg
Sample desorption temperature	350 °C
Desorption time	7 min
Carrier gas flow during tube desorption	60 mL/min
Carrier gas flow during trap desorption	1.0 mL/min
Trap temperature	−20 °C
Trap desorption temperature	350 °C
Desorption time	3 min
Valve, transfer line and injection port temperature	300 °C

**Table 3:** Operating parameters of the GC/MS chromatograph.

Working Parameter	Characteristic
Injection temperature	300 °C
Splitless time	1 min
Carrier gas	Helium 0.92 mL/min, constant flow, linear velocity 25.8 cm/s
Temperature	Isothermal program 60 °C for 1 min, then increase at a rate of 15 °C/min to 320 °C, maintained for 12 min
Ion Source Temp.	300 °C
Interface temp	300 °C
Detector voltage	0.7 kV
Detector mode	SCAN 35–300 amu

**Table 4:** Results of analyses of special clothing (shoes, trousers, jacket, gloves) for the presence of PAH compounds (in µg/g).

Analyte	Toe of Shoe	Shoe Insole	Knee from the Front	Knee from the Back	Thigh from the Front	Crotch	Buttock	Inner Cuff	Armpit	Chest in Front	Back at Shoulder Blade Height	Inner Collar Cuff	Inner Cuff from Forearm	Gloves—Outside	Gloves—Inner Side
Naphthalene	<0.02	0.13 ± 0.01	0.15 ± 0.01	0.10 ± 0.01	0.16 ± 0.01	0.09 ± 0.01	0.09 ± 0.01	0.06 ± 0.01	0.06 ± 0.01	0.07 ± 0.01	0.06 ± 0.01	0.06 ± 0.01	<0.02	1.42 ± 0.04	<0.02
Acenaphthylene	0.02 ± 0.01	<0.02	<0.02	<0.02	0.02 ± 0.01	<0.02	0.03 ± 0.01	<0.02	<0.02	<0.02	<0.02	<0.02	<0.02	0.10 ± 0.01	<0.02
1-methylnaphthalene	0.51 ± 0.04	0.65 ± 0.05	0.62 ± 0.05	0.06 ± 0.01	0.40 ± 0.03	0.41 ± 0.03	<0.02	0.09 ± 0.01	0.06 ± 0.01	0.17 ± 0.01	0.09 ± 0.01	0.09 ± 0.01	<0.02	<0.02	<0.02
2-methylnaphthalene	0.26 ± 0.02	0.03 ± 0.01	<0.02	<0.02	0.02 ± 0.01	2.11 ± 0.17	<0.02	0.41 ± 0.03	0.46 ± 0.04	0.03 ± 0.01	0.41 ± 0.03	0.08 ± 0.01	<0.02	0.15 ± 0.01	<0.02
Acenaphthene	0.02 ± 0.01	<0.02	0.04 ± 0.01	0.04 ± 0.01	0.03 ± 0.01	0.03 ± 0.01	0.02 ± 0.01	0.02 ± 0.01	0.03 ± 0.01	0.04 ± 0.01	0.02 ± 0.01	<0.02	<0.02	0.27 ± 0.01	<0.02
Fluorene	0.21 ± 0.01	0.59 ± 0.02	0.23 ± 0.01	0.19 ± 0.01	<0.02	0.13 ± 0.01	0.10 ± 0.01	0.05 ± 0.01	0.05 ± 0.01	0.11 ± 0.01	0.05 ± 0.01	<0.02	<0.02	0.91 ± 0.03	<0.02
Phenanthrene	<0.02	0.30 ± 0.03	0.22 ± 0.02	0.16 ± 0.02	0.17 ± 0.02	0.23 ± 0.03	0.10 ± 0.01	0.50 ± 0.06	1.13 ± 0.13	1.05 ± 0.12	0.50 ± 0.06	0.15 ± 0.02	<0.02	1.46 ± 0.16	<0.02
Anthracene	<0.02	0.29 ± 0.01	0.21 ± 0.01	<0.02	0.16 ± 0.01	0.20 ± 0.01	<0.02	0.59 ± 0.03	1.10 ± 0.05	1.01 ± 0.05	0.59 ± 0.03	<0.02	<0.02	1.40 ± 0.07	<0.02
Fluoroantennas	0.09 ± 0.01	0.03 ± 0.01	0.05 ± 0.01	0.04 ± 0.01	0.03 ± 0.01	0.04 ± 0.01	<0.02	0.05 ± 0.01	0.03 ± 0.01	0.13 ± 0.01	0.05 ± 0.01	<0.02	<0.02	<0.02	<0.02
Pyrene	0.09 ± 0.01	0.09 ± 0.01	<0.02	0.04 ± 0.01	<0.02	0.03 ± 0.01	0.02 ± 0.01	0.04 ± 0.01	0.05 ± 0.01	0.12 ± 0.01	0.04 ± 0.01	0.04 ± 0.01	<0.02	0.02 ± 0.01	<0.02
Benzo(a)anthracene	0.07 ± 0.01	0.06 ± 0.01	0.03 ± 0.01	0.03 ± 0.01	0.03 ± 0.01	0.03 ± 0.01	<0.02	0.03 ± 0.01	0.02 ± 0.01	0.05 ± 0.01	0.03 ± 0.01	<0.02	<0.02	<0.02	<0.02
Chrysene	<0.02	0.07 ± 0.01	<0.02	<0.02	0.02 ± 0.01	0.06 ± 0.01	0.03 ± 0.01	0.02 ± 0.01	0.02 ± 0.01	0.05 ± 0.01	0.02 ± 0.01	0.02 ± 0.01	<0.02	0.02 ± 0.01	<0.02
Benzo(b)fluoroanthenes	<0.02	0.06 ± 0.01	0.03 ± 0.01	<0.02	0.03 ± 0.01	0.03 ± 0.01	<0.02	<0.02	<0.02	<0.02	<0.02	0.04 ± 0.01	<0.02	<0.02	<0.02
Benzo(k)fluoroantennas	<0.02	0.12 ± 0.01	0.05 ± 0.01	0.23 ± 0.02	0.11 ± 0.01	0.06 ± 0.01	<0.02	0.02 ± 0.01	0.09 ± 0.01	0.03 ± 0.01	0.02 ± 0.01	0.04 ± 0.01	<0.02	0.08 ± 0.01	<0.02
Benzo(a)pyrene	<0.02	0.04 ± 0.01	<0.02	<0.02	0.02 ± 0.01	0.02 ± 0.01	<0.02	<0.02	<0.02	<0.02	<0.02	0.05 ± 0.01	<0.02	<0.02	<0.02
Dibenzo(a.h)anthracene	<0.02	0.02 ± 0.01	<0.02	<0.02	0.02 ± 0.01	<0.02	<0.02	<0.02	<0.02	<0.02	<0.02	<0.02	<0.02	<0.02	<0.02
Benzo(g.h.i)perylene	<0.02	0.06 ± 0.01	0.02 ± 0.01	<0.02	<0.02	0.03 ± 0.01	<0.02	<0.02	<0.02	<0.02	<0.02	<0.02	<0.02	<0.02	<0.02
Indeno(1.2.3-c.d)pyrene	<0.02	0.02 ± 0.01	<0.02	<0.02	<0.02	<0.02	<0.02	<0.02	<0.02	<0.02	<0.02	<0.02	<0.02	<0.02	<0.02
Total PAHs	5.43	1.43	2.58	1.68	0.96	1.23	3.50	0.44	3.11	2.90	1.90	0.57	<0.02	5.86	<0.02

Yellow color—zone I, green color—zone II, purple color—zone III, orange color—zone IV.

**Table 5:** The order of sorption of individual compounds from the PAH group on the tested materials in relation to the properties of the analytes (data characterizing PAHs based on [61]).

Chemical	Order of Sorption	Molecular Weight	Boiling Point	Diffusivity in Air	Enthalpy of Vaporization at Normal Boiling Point	Henry’s Law Constant	Water Solubility	Vapor Pressure
		[g/mol]	[°C]	[cm^2^/s]			[mg/dm^3^]	[mm Hg]
Low Molecular Weight PAHs								
Naphthalene	g > s,t,j	128.18	217.9	6.05 × 10^−2^	10,325.0478	4.40 × 10^−4^	31	8.50 × 10^−2^
Methylnaphthalene, 1-	s > j,t > g	142.2	244.7	5.28 × 10^−2^	10,874.76099	5.14 × 10^−4^	25.8	6.70 × 10^−2^
Methylnaphthalene, 2-	j,t > s,g	142.2	241.1	5.24 × 10^−2^	12,600	5.18 × 10^−4^	24.6	5.50 × 10^−2^
Acenaphthylene	s,t,j,g	152.2	280	4.50 × 10^−2^	11,714.62714	1.14 × 10^−4^	16.1	6.68 × 10^−3^
Acenaphthene	g > s,t,j	154.21	279	5.06 × 10^−2^	12,155	1.84 × 10^−4^	3.9	2.15 × 10^−3^
Fluorene	s > g > j,t	166.22	295	4.40 × 10^−2^	12,666	9.62 × 10^−5^	1.69	6.00 × 10^−4^
Anthracene	j,t > g > s	178.24	339.9	3.90 × 10^−2^	13,121	5.56 × 10^−5^	4.34 × 10^−2^	6.53 × 10^−6^
Phenanthrene		178.24	340	3.45 × 10^−2^	12,915.15296	4.23 × 10^−5^	1.15	1.21 × 10^−4^
High Molecular Weight PAHs								
Fluoranthene	s,j > t > g	202.26	384	2.76 × 10^−2^	13,757.17016	8.86 × 10^−6^	0.26	9.22 × 10^−6^
Pyrene	s,j > t > g	202.26	404	2.78 × 10^−2^	14,370	1.19 × 10^−5^	0.135	4.50 × 10^−6^
Benz[a]anthracene	s,j > t > g	228.3	437.6	2.61 × 10^−2^	16,000	1.20 × 10^−5^	9.40 × 10^−3^	2.10 × 10^−7^
Chrysene	j,t > s,g	228.3	448	2.61 × 10^−2^	16,467.49521	5.23 × 10^−6^	2.00 × 10^−3^	6.23 × 10^−9^
Benzo[a]pyrene	s,t,j > g	252.32	495	2.55 × 10^−2^	14,412.52389	4.57 × 10^−7^	1.62 × 10^−3^	5.49 × 10^−9^
Benzo[b]fluoranthene	s > j,t > g	252.32	442.75	2.50 × 10^−2^	14,412.52389	6.57 × 10^−7^	1.50 × 10^−3^	5.00 × 10^−7^
Benzo[j]fluoranthene	s > j,t > g	252.32	480	2.50 × 10^−2^	16,412.04588	5.84 × 10^−7^	8.00 × 10^−4^	9.65 × 10^−10^
Benzo[g,h,i]perylene	s,t,j > g	276.34	486.31	2.39 × 10^−2^	17,747.60993	3.31 × 10^−7^	2.60 × 10^−4^	1.00 × 10^−10^
Indeno[1,2,3-cd]pyrene	s,t,j > g	276.34	536	2.47 × 10^−2^	17,747.60993	3.48 × 10^−7^	1.90 × 10^−4^	1.25 × 10^−10^
Dibenz[a,h]anthracene	s,t,j > g	278.36	524	2.36 × 10^−2^	17,341.30018	1.41 × 10^−7^	2.49 × 10^−3^	9.55 × 10^−10^

Where s—shoes (rubber); j—jacket (aramid fiber, antistatic fiber, aramid fiber covered with a polyethylene film, viscose layer); t—trousers (aramid fiber, antistatic fiber, aramid fiber covered with a polyethylene film, viscose layer); g—gloves (full-grain cowhide, HIPORA polyurethane membrane, inner insert made of Kevlar fibers).

**Table 6:** Comparison of the average contents of selected analytes in the sample and firefighter’s clothing with PEL and RD values.

Analyte	Average Content in the Sample (μg/g)	Estimated Contents in Whole Clothes (μg)	PEL (μg/m^3^)	Respirator’s Daily Intake Max (μg)
Naphthalene	0.16	1155	100	4656
Phenanthrene	0.40	2797	8.88	413
Anthracene	0.37	2605	0.79	37
Pyrene	0.04	271	9.00	419
Chrysene	0.02	164	3.27	152
Benzo(a)pyrene	0.01	80	2.49	116

## Data Availability

Data are contained within the article.
